# Effects of thymoquinone in prevention of experimental contrast-induced nephropathy in rats

**DOI:** 10.22038/IJBMS.2019.13990

**Published:** 2019-12

**Authors:** Ulaş Serkan Topaloğlu, Murat Hayri Sipahioğlu, İnayet Güntürk, Hülya Akgün, Muhammet Ensar Doğan, Gökhan Sönmez, Ferhan Elmalı, Cevat Yazıcı

**Affiliations:** 1 Department of Internal Medicine, Kayseri City Hospital, Kayseri, Turkey; 2 Department of Nephrology, Erciyes University Medical Faculty, Kayseri, Turkey; 3 Department of Medical Biochemistry, Ömer Halisdemir University School of Medicine, Niğde, Turkey; 4 Department of Pathology, Erciyes University Medical Faculty, Kayseri, Turkey; 5 Department of Medical Genetics, Erciyes University Medical Faculty, Kayseri, Turkey; 6 Department of Urology, Kayseri City Hospital, Kayseri, Turkey; 7 Department of Biostatistics, İzmir Katip Çelebi Üniversitesi Medical Faculty, İzmir, Turkey; 8 Department of Biochemistry, Erciyes University Medical Faculty, Kayseri, Turkey

**Keywords:** Contrast-induced-nephropathy, Inflammation, Oxidative stress, Nigella sativa, Rat, Thymoquinone

## Abstract

**Objective(s):**

This study aimed to show the effects of thymoquinone, which is known for its antioxidant, anti-inflammatory, and renal protective effects in contrast-induced nephropathy.

**Materials and Methods:**

This is an experimental study in rats. 7 groups were included within the scope of our study: sham-vehicle (n=3), premedication-control (n=6), model (n=6), isolated thymoquinone (n=3+3), low-dose thymoquinone (n=6), and high-dose thymoquinone (n=7). In addition to 48 hr of water deprivation, we pre-medicated the rats with intra-peritoneal indomethacin and L-NAME administration. After premedication, 12.5 ml/kg dose of a high osmolar contrast agent-diatrizoat (Urografin %76) was administrated. Thymoquinone was administrated in two different doses of 1 mg/kg and 1.75 mg/kg for four days intraperitoneally. Renal functions, histopathological differences, oxidative stress parameters, and inflammatory indicators of rats were evaluated at the end of the study.

**Results:**

Significant decreases were observed in levels of serum creatinine and serum BUN with low-dose thymoquinone (1 mg/kg) administration. In light microscopy, significantly less histopathological damage was observed in the low-dose thymoquinone group compared to the contrast agent group. While high-dose thymoquinone is accepted as ineffective biochemically, toxic evidence was identified histopathologically. There were no significant differences between M and TA groups for serum MDA and SOD levels, which were compared to evaluate oxidative stress (*P:*0.99, *P:*0.98; respectively). TNF-α, iNOS, and NF-кB gene expressions were not significantly different between all groups (*P:*0.748, *P:*0.531, *P:*0.910; respectively).

**Conclusion:**

This experimental study has demonstrated for the first time the protective effect of the TQ substance for CIN in 1 mg/kg dose, in the accompaniment of biochemical and histopathological data in rats.

## Introduction

Contrast-induced nephropathy (CIN) is an acute kidney injury (AKI) developing after intravenous (IV) or intraarterial use of contrast media (CM) (1). Though generally reversible, it may be associated with adverse outcomes. In practice, it is increasingly confronted as a more common clinical problem in line with the gradual increase in the use of CM. The most effective method known today in protection against CIN development is to provide appropriate hydration to the patient (2).

The pathophysiology of CIN could not yet be adequately clarified. Renal medullar hypoxia resulting from reduction in blood flow associated with renal artery vasoconstriction, direct tubular toxicity caused by CM through apoptosis and oxidative stress, endothelial dysfunction, and changes in renal microcirculation are the factors thought and considered to be effective in pathogenesis (3, 4).

*Nigella Sativa (N. Sativa)* is a kind of yearling herbaceous plant from the *Ranunculaceae* family. In traditional medicine, *N. Sativa* seeds are used for therapeutic purposes in such conditions as coughing, high fever, asthma, hypertension, diabetes, and eczemas (5, 6). Thymoquinone (2-isopropyl-5-methyl-1,4-benzoquinone) is a compound obtained from *N. Sativa* oil. A great many clinical studies have so far revealed anti-oxidant, anti-inflammatory, anti-cancer, hepatoprotective, and renoprotective characteristics of thymoquinone (TQ) (7). In studies performed by using cell culture, it is recorded that some genes such as nuclear factor kappa B (NF-ĸB), being a proinflammatory transcription factor, and interleukin-6 (IL-6) controlled by it, are suppressed by TQ, and thus, it protects renal tubular cells (8). Harmful effects of free oxygen radicals (FOR) may be prevented through stimulation of neutrophils by TQ (9). TQ leads to reduction of enzyme activities such as superoxide dismutase (SOD), catalase, and glutathione peroxidase (10).

Departing from the aforesaid characteristics of TQ, and the mechanisms effective in CIN pathophysiology, the effects of TQ in the experimental CIN model are investigated in this study. 

According to the results of our literature screening (PubMed, Google academic), this study is the first in medical literature investigating the efficiency of TQ prophylaxis in prevention of development of nephropathy in the CIN model.

**Table 1:** Diagram of application of experiment by groups and days

	**S**	**P**	**M**	**T1, T2**	**TA, TB**
Pre-trial	Compliance				
0 hour	DMSO	DMSO	DMSO	TQ	TQ
8^th^ hour	water is free	water is restricted	water is restricted	water is free	water is restricted
24^th^ hour	DMSO	DMSO	DMSO	TQ	TQ
40+8^th^ hour	DMSO DMSO SF SF	DMSO INDO L-NAME SF	DMSO INDO L-NAME UROGRAFIN	TQ DMSO SF SF	TQ INDO L-NAME UROGRAFIN
48+8^th^ hour	water is freely supplied to all groups				
72^nd^ hour	DMSO	DMSO	DMSO	TQ	TQ
96^th^ hour	SACRIFICATION				

S: Sham-vehicle group, P: Premedication-control group, M: Model group with contrast media, T1 and T2: Naked TQ groups, TA: CM+TQ_1_ group, TB: CM+TQ_1.75_ group; DMSO: Dimethyl sulfoxide, TQ: Thymoquinone, SF: Saline fluid, INDO: Indomethacin, L-NAME: N(ω)-nitro-L-arginine methyl ester

**Table 2:** Intergroup renal function tests and antioxidant indicators

	S (n=3)	P (n=3)	M (n=5)	T1 (n=3)	T2 (n=3)	TA (n=5)	TB (n=5)	P
SCre (mg/dl)	0,30±0,03	0,56±0,27^a^	2,03±1,28^a,b^	0,29±0,01	0,28±0,02	0,41±0,06^b,c^	2,23±0,80^c^	a=0,008 b=0,004 c=0,002
Serum BUN (mg/dl)	20,7±1,4	67,5±64,7^a^	179,7±98,7^a,b^	17,6±1,9	16,2±1,8	21,9±9,4^b,c^	210,3±62,4^c^	a=0,013 b<0,001 c<0,001
Serum NGAL (ng/ml)	13,63±1,26	6,91±3,49	12,07±3,36	9,79±3,02	9,71±2,23	10,52±2,88	10,79±4,68	NS
Serum MDA (µmol/L)	7,74±2,62^a^	20,37±9,06^a,b,c^	5,71±3,39^b^	5,58±1,99	6,56±1,34	7,13±3,18^c^	7,00±3,28	a=0,001 b<0,001 c<0,001
Serum SOD (U/mL)	22,00±0,93	15,80±2,72	20,74±4,91	21,64±1,47	21,22±2,33	20,89±2,10	19,79±3,49	NS

*P<*0.05 is considered as significant. Statistically significant *P-values* are shown as a,b, and c. NS: Not statistically significant.

S: Sham-vehicle groupa, P: Premedication-control group, M: Model group with contrast media, T1 and T2: Naked TQ groups, TA: CM+TQ_1_ group, TB: CM+TQ_1.75_ group; Scre: Serum creatinine, BUN: Blood urea nitrogen, NGAL: neutrophil gelatinase-associated lipocalin , MDA: malondialdehyde , SOD: superoxide dismutase

**Table 3:** Intergroup anti-inflammatory parameters

	**iNOS**	**NF-кB**	**TNF-α**
**S**	0.01320 ± 0.000781	0.01 ± 0.002	0.0006 ± 0.00005
**P**	0.01443 ± 0.002685	0.01 ± 0.002	0.0006 ± 0.00011
**M**	0.01446 ± 0.002485	0.02 ± 0.002	0.0008 ± 0.00054
**T1**	0.01263 ± 0.001124	0.02 ± 0.007	0.0004 ± 0.00004
**T2**	0.01210 ± 0.002100	0.01 ± 0.001	0.0005 ± 0.00008
**TA**	0.15880 ± 0.341735	0.10 ± 0.207	0.0752 ± 0.16711
**TB**	0.05324 ± 0.084679	0.01 ± 0.009	0.0031 ± 0.00438
**P**	**NS**	**NS**	**NS**

NS: Not statistically significant. S: Sham-vehicle group, P: Premedication-control group, M: Model group with contrast media, T1 and T2: Naked TQ groups, TA: CM+TQ_1_ group, TB: CM+TQ_1.75_ group

**Table 4:** Intergroup histopathological assessment

Trial group	Rat no	Tubular necrosis	*P*	Proteinous cast	*P*	Medullary congestion	*P*
S, P, T1 (n=3,3,3)	1	0	a	0	x	0	Α
	2	0		0		0	
	3	0		0		0	
T2 (n=3)	1	0	a	0	x	1	Β
	2	0		0		1	
	3	0		0		1	
M (n=3)	1	1	b	1	y	1	Β
	2	2		1		1	
	3	2		2		2	
	4	1		1		1	
	5	2		1		1	
TA (n=5)	1	0	a	0	x	1	Β
	2	0		0		1	
	3	0		0		1	
	4	0		0		1	
	5	0		0		1	
TB (n=5)	1	1	c	1	y	1	Β
	2	2		1		1	
	3	4		2		2	
	4	3		1		1	
	5	3		2		1	

*P<*0.05 is considered and treated as significant. In *P-values*, there are significant differences among groups marked with different letters.

S: Sham-vehicle group, P: Premedication-control group, M: Model group with contrast media, T1 and T2: Naked TQ groups, TA: CM+TQ_1_ group, TB: CM+TQ_1.75_ group

**Figure 1:**
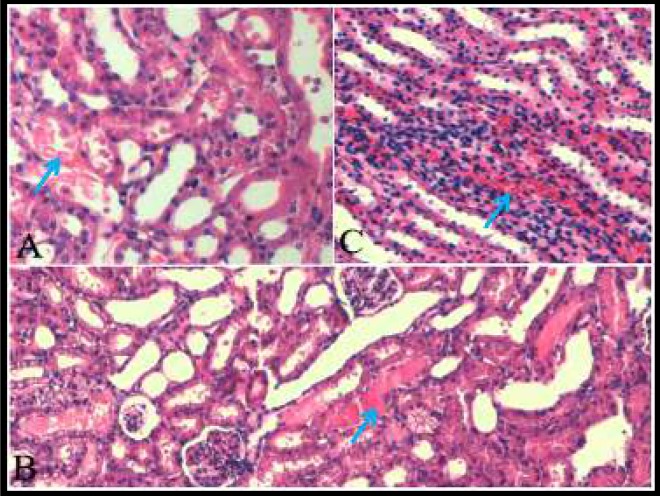
Histopathological views of nephropathy induced and generated by contrast media

A: Tubular necrosis (HE x200). B: Proteinous cast (HE x100). C: Medullary congestion (HE x100)

## Materials and Methods

***Animals***

This is an experimental study in rats. 34 Wistar albino type adult male rats of 20 weeks of age, with a bodyweight between 250 and 350 g, grown in Erciyes University Experimental and Clinic Study Center were included in this study. The number of rats was determined through a pre-study power analysis. Throughout the study, rats were hosted under equal conditions in terms of temperature (22±3 ^°^C), humidity (55–60%) and light (12 hr light, 12 hr darkness), and throughout the study, rats were fed standard rat diet *ad libitum*. The time of the initial 24 hr after grouping was determined and used as the orientation time of rats with the new environment. Their bodyweights were calculated at the beginning and end of the experiment. Rats in all groups were sacrificed at the 96^th^ hr of the study. 

In order to minimize the probable effects of circadian rhythm, the weighing, medication, sacrification, blood, and renal sampling operations were conducted at 09:00 on a daily basis. Before sacrification, for anesthesia purposes, 50 mg/kg ketamine (Ketalar Vial, Pfizer, Istanbul, Turkey) and 10 mg/kg xylazine (Rompun Vial, Bayer, Istanbul, Turkey) were intraperitoneally (IP) administered to rats. The experiment was finished by taking blood samples through intracardiac injection and by removal of both kidneys from rats. 

***Chemicals***

Dimethyl sulfoxide (DMSO): Procured from Bioshop firm (catalogue no.: DMS555.500), of >99% purity. Used in order to dissolve TQ and indomethazine (INDO) substances. It was administered in equal amount to all rats, and totally 1500 mg/kg was given to each rat throughout the study.

Thymoquinone: Procured from Sigma-Aldrich firm (catalogue no.: 274666-1G), of >99% purity. Before the experiment, it was dissolved in DMSO, and stored in a dark environment at -20 ^°^C temperature. 

N(ω)-nitro-L-arginine methyl ester (L-NAME): Procured from Sigma-Aldrich firm (catalogue no.: N5751-1G), of >98% purity. It was dissolved in saline fluid (SF) before administration to rats.

Indomethacin: Procured from Sigma-Aldrich firm (catalogue no.: I7378-5G), of >99% purity. It was dissolved in DMSO before administration to rats.

Meglumine/sodium diatrizoate (Urografin 76%): Procured from Bayer-Schering firm, and contained sodium amidotrizoate 0.1 g/ml, meglumine amidotrizoate 0.66 g/ml and iodine 370 mg/ml.

***Creation of contrast nephropathy model:***

In order to create the CIN model, a predisposing effect was generated without causing nephropathy. This effect was provided by dehydration of 48 hr, 10 mg/kg L-NAME, and 10 mg/kg INDO. As CM, 12.5 ml/kg meglumine/sodium diatrizoate was used. Dosages of these agents were determined and decided on the basis of the existing literature data (11-14) and the results of the preliminary experiment conducted by us prior to the study. INDO, L-NAME, and CM were administered via the IP route at the 40^th^ hr of dehydration with time intervals of 30 min. After completion of these steps, rats were deprived of water for a further 8 hr to make their kidneys more sensitive to CM. 

***Determination of thymoquinone dosage:***

A literature review revealed that doses used for TQ administered via the IP route are within a very wide range (0.5–80 mg/kg) (15-17). As in a rat study*,* 8 mg/kg dose has been determined to be toxic, by using that study as a reference, 2.5 mg/kg and 5 mg/kg doses were chosen for a preliminary experiment (16). Said doses demonstrated a nephrotoxic effect in the preliminary experiment. Considering the literature and preliminary results, we determined the dosage of TQ as 1 mg/kg and 1.75 mg/kg, respectively. TQ administration is effected 30 min before INDO administration. 

***Study groups***

Sham-vehicle group (S, n=3): Dehydration was not applied in this group created solely to observe and determine the effects of solvents. Rats in this group were administered DMSO instead of TQ once at each of 0^th^, 24^th^, 48^th^, and 72^th^ hr, and in addition, in the 3^rd^ day of the experiment, DMSO instead of INDO, and SF instead of L-NAME and SF were administered in equal volumes and frequency.

Premedication-control group (P, n=6): These rats were exposed to dehydration during the initial 48 hr of study. These rats were administered DMSO instead of TQ in equal volumes and frequency once at each of 0^th^, 24^th^, 48^th^, and 72^nd^ hr. INDO and L-NAME were given at the 40^th^ hr of dehydration.

The model group with contrast media (M, n=6): These rats were exposed to dehydration during the initial 48 hr of study. They were administered DMSO instead of TQ in equal volumes and frequency once at each of 0^th^, 24^th^, 48^th^, and 72^nd^ hr. INDO, L-NAME, and CM were given at the 40^th^ hr of dehydration.

Naked TQ groups (T1 and T2, n=3 for each group): In this group created for observation of naked TQ effects, TQ was administered to rats once at each of 0^th^, 24^th^, 48^th^, and 72^nd^ hr. TQ doses were determined as 1 mg/kg in T1 group (n=3) and 1.75 mg/kg in T2 group (n=3). Water supply was not restricted in this group. After administration of TQ to rats at the 48^th^ hr of study, unlike other groups, DMSO instead of INDO, and SF instead of L-NAME and CM were given to rats of this group in equal volumes and frequency.

CM+TQ_1_ group (TA, n=6): These rats were exposed to dehydration during the initial 48 hr of study. These rats were administered 1 mg/kg TQ dose once at each of 0^th^, 24^th^, 48^th^, and 72^nd^ hr. INDO, L-NAME, and CM were given 30 min after administration of TQ at the 40^th^ hr of dehydration.

CM+TQ_1.75_ group (TB, n=7): Differently from CM+TQ_1_ group, TQ dosage was determined as 1.75 mg/kg.

All of these groups and administrations are summarized in Table 1.

***Determination of Renal Functions***

Serum creatinine (Scre) and serum BUN (blood urea nitrogen) were measured using an automatic biochemical analyzer (Roche Hitachi Cobas 8000).

Neutrophil gelatinase-associated lipocalin (NGAL): By using Rat-NGAL ELISA kit (catalogue no. 201-11-1763) procured from Sunred firm, China; serum samples were processed manually by the sandwich enzyme immunoassay method.

***Determination of Oxidative Stress Level***

Malondialdehyde (MDA) was measured fluorometrically over plasma between 530 and 550 nm wavelengths by using Cayman’s TBARS Assay kit (catalogue no.: 10009055).

Superoxide dismutase (SOD) activity was determined spectrophotometrically by using Cayman’s Superoxide Dismutase Assay kit (catalogue no.: 706002). 

***Determination of inflammatory levels***

NF-кB, tumor necrosis factor-α (TNF-α) and inducible nitric oxide synthase (iNOS) gene expression levels were determined by RNA insulation, cDNA yield, and RT-PCR method over left kidney removed from rats.

***Renal histopathology***

After sacrification, the right kidney was removed and fixed in 10% formalin. After that, it was embedded in paraffin blocks, and 4 μm cross-sections were taken and dyed by Hematoxyline & Eosin (H&E). Then, preparations were examined using optical light microscopy by a single pathologist. Tubular necrosis (TN), proteinous cast (PC) and medullary congestion (MC) conditions were scored between 0 and 4 depending on the degrees of damages there in (18-20). 

***Statistical analysis***

For statistical analyses, SPSS 22.0 (IBM Corp, Armonk, NY, USA) program was used. Whether numeric (digital) data was distributed normally or not was determined by Shapiro-Wilks test and Histogram graphs. Numeric (digital) data relating to independent groups demonstrating a normal distribution were compared using One-Way ANOVA test, and *Post-Hoc* analyses of the test were performed by Tukey’s test. For comparison of categorical data, the Chi-square test was used. Ordinal data relating to multiple independent groups were analyzed by the Kruskal-Wallis test, and for *post hoc* analysis of this test, Bonferroni test was employed. All continuous variables were expressed in average±standard deviation units, while frequencies were expressed in percentage (%). *P*<0.05 was accepted and considered as significant. 

## Results

During the study, three rats from group P, one rat from group M, one rat from group TA, and two rats from group TB lost their lives one day before sacrification.

As for bodyweights of rats before and after the experiment, no significant difference was detected and determined between the groups (*P*>0.05).

***Renal functions, antioxidant and antiinflammatory parameters***

In intergroup assessments regarding renal function tests, it was determined that SCre levels measured at the end of the experiment were significantly higher in groups M and TB in comparison to other groups (respectively; *P*:0.008, *P*:0.002). And there was no difference between groups TB and M (*P*>0.05). Serum BUN levels measured at the end of the experiment also varied similarly to SCre levels (respectively; *P*:0.013, *P*<0.001) (Table 2).

A review of NGAL levels being the indicator of renal functions, and serum MDA and SOD levels being the antioxidant indicators, and expression values of iNOS, NF-кB, and TNF-α genes measured in renal tissue and being among anti-inflammatory parameters revealed no significant difference among groups at the end of the experiment. The data of all these parameters are shown in Tables 2 and 3. Some statistical differences that were significant in MDA results do not contribute to the study. There were no significant differences between M and TA groups for serum MDA and SOD levels, which were compared to evaluate oxidative stress (respectively; *P*:0.99, *P*:0.98). TNF-α, iNOS, and NF-кB gene expressions were not significantly different between all groups (respectively; *P*:0.748, *P*:0.531, *P*:0.910).

***Histopathological findings***

A review of TN and PC in histopathological damage assessment revealed no TN in groups S, P, T1, T2, and TA, while damages of varying degrees were detected in M and TB groups. Degree of these damages was significantly higher than other groups (respectively; *P*<0.05, *P*<0.001). A comparison between M and TB groups revealed more damages in group TB in terms of TN, while there was no significant difference between these two groups in terms of PC (respectively; *P*<0.05, *P*>0.05) (Table 4).

MC was also examined, and no damage was detected in S, P, and T1 groups, while damages of varying degrees were detected in M, T2, TA, and TB groups. Levels of damages in these groups were significantly higher in comparison to groups with no damage observation (*P*<0.001). An assessment of groups with MC damage observation among themselves revealed no statistically significant difference (*P*>0.05) (Table 4).

Pathological views of nephropathy, induced and generated by contrast media, are shown in Figure 1.

## Discussion

Just like healthy humans, also in experimental models, administration of CM does not alone lead to nephropathy in animals not bearing any predisposing factors (21). Nephropathy could be created very difficultly, and could not even be created in some studies, even by leaving rats dehydrated for a certain period of time and/or by injecting furosemide and/or by applying unilateral nephrectomy for predisposition before administration of CM (22–24). In a rat study included in the medical literature, it is reported that CM administered after dehydration for 72 hr could not create nephropathy. Again in the same study, rats left dehydrated for 48 hr were administered CM after furosemide injection, but the model could not be created (25).

Prostaglandin (PG) and NO give medullary oxygen support by increasing regional blood flow. If and when PG and NO synthesis is suppressed respectively by nonsteroidal anti-inflammatory drugs and L-NAME, a predisposition may be created for CIN. By departing from this interpretation, a fairly successful CIN model was created (11). In another model*,* in addition to administration of INDO and L-NAME, rats were also dehydrated for 24 hr for predisposition purpose. At the end of the experiment, it was noted that serum BUN and SCre values in the CIN model group were increased by more than two folds in comparison with the control group (13) . The preliminary experiment conducted by us demonstrated that the CIN model was not created when CM was given in the dose (7 ml/kg) specified in the medical literature, and INDO (10 mg/kg) and L-NAME (10 mg/kg) are administered for predisposing effect. Thereupon, dehydration of 48 hr was added to the model as an additional predisposition, and the CM dose was also increased (10 ml/kg and 14 ml/kg). In the repeated experiments, administration of CM in 10 ml/kg dose did not create the model, while administration in 14 ml/kg dose led to a fatal impact for half of the rats. Upon these test results, a new model was created for rats, and the dose of urography to be applied was determined as 12.5 ml/kg with a reduction from 14 ml/kg dose determined by us and accepted and treated as LD 50. When CM was administered in the dose above, it was also confirmed by histopathological findings that the CIN model was created, and SCre and serum BUN levels increased.

In clinical practice, increase in SCre levels may also be used in AKI and CIN diagnoses. However, SCre level was inadequate in demonstrating the changes occurring in the acute period because it is affected from such factors as patient’s age, gender, muscular mass, and hydration status, and it is probable not to record any evident rise until renal functions decrease by 50%; it incurs tubular secretion at low glomerular filtration rate levels, and its rise is completed in a rather long time (26). NGAL is an extracellular protein belonging to the lipocalin family (27). It is reported that as a result of apoptosis stimulated by oxidative stress and complement, damaged renal tubular cells lead to NGAL production (28). In the medical literature, there are studies defending serum and urine NGAL levels measured in the early period and particularly within the initial 4 hr is effective in CIN diagnosis (29–33). In our study, we found no statistically significant difference among groups in terms of serum NGAL levels measured at the end of the experiment. However, in our study, blood samples were taken 48 hr after CM exposure. We think that these results have been derived out of our study because of this rather long duration and because NGAL levels were measured only in serum in our study. 

The medical literature includes a great many clinic studies where TQ is administered orally or via the IP route in non-CIN nephropathy models. In gentamicin-induced nephropathy (34), doxorubicin-induced nephrotic syndrome (35), mercury chloride-induced renal toxicity (17), ifosfamide-induced nephropathy (36), and cisplatin-induced nephropathy (37) models, TQ is demonstrated to be efficient when administered orally. While in administration via the IP route, there are clinical studies defending its antioxidant and antiinflammatory efficacy (38), and its efficiency in secondary nephropathy (39) and nephropathy secondary to arthritis (40). Taking into consideration that orally administered TQ may not reach its efficient dose and may not adequately be dissolved when taken with drinking water, the aspiration risk, and that in case of vomiting, the administration remains below the desired and targeted dosage, and the disadvantages of oral bioavailability, we decided in our study that more effective outcomes may be achieved through administration via the IP route.

In a rat nephrotoxicity model related to acetaminophen, a significant reduction is reached in SCre and serum BUN values when 10 mg/kg oral TQ is administered to rats (41). It was demonstrated that 2 mg/kg TQ via the IP route creates an improvement in SCre and BUN levels in rats in which arthritis and renal damages are induced by Pristina (40). In a study for renal impairment induced by gentamicin, TQ is administered via the IP route, and a significant improvement is demonstrated in SCre and BUN levels (42). In our study, coherently with the medical literature, it is observed that 1 mg/kg dose of TQ led to a dramatic improvement in SCre and serum BUN levels in rats in which a CIN model is created, while the 1.75 mg/kg dose resulted in a worsening in these values. These results give rise to the thought that TQ exerts its protective effect only when it is administered in the appropriate dose, but may further increase toxicity when it is administered in higher doses together with CM.

In a nephropathy rat model generated by unilateral urethral obstruction, the efficacy of 10 mg/kg dose of TQ administered via the IP route has been compared to that of captopril and losartan known as renin-angiotensin system inhibitors (39). A review of the comparison results reveals that in therapy groups, MDA and TNF-α levels processed on renal tissue reduced, while SOD levels increased. Based on these results, the authors concluded and defended that TQ is as efficient as captopril and losartan. It is thought to have the curative effect of thymoquinone and is able to improve the renal tissue damage like them. A group of researchers has administered 2 mg/kg TQ via the IP route to rats in which arthritis and renal damages are induced and generated by the Pristina substance. At the end of this clinical study, it was demonstrated that TQ procured an improvement in anti-inflammatory parameters (40). In another clinical study where nephrotoxicity was induced by gentamicin, TQ’s anti-inflammatory and antioxidant efficacy was demonstrated through MDA and TNF-α (42). In patients with diabetes, through *in vitro* glycolyzation of proximal renal tubular epithelium cells, which are target cells of hyperglycemia, NF-ĸB activation is created, and TQ efficiency is studied in the resulting inflammation therein. The authors have inhibited NF-ĸB activation of TQ and indicated its protective effects on renal tubular cells (8). In a hepatotoxicity model generated and induced by cisplatin, the mechanism of benefits of TQ used for prevention of toxicity is described, and it is emphasized that TQ is efficient not only through MDA, TNF-α, and NF-ĸB pathways but also through iNOS. It is further demonstrated that iNOS expression increased by cisplatin is reduced by TQ administration. It was concluded that TQ has potential benefits in the prevention of the onset and progression of cisplatin-induced hepatotoxicity through these ways (43). In our study, it is found out that TQ does not exert any effect on MDA and SOD levels, which are among oxidative stress parameters. We think that this result may have been caused by our studying MDA and SOD levels in serum samples, rather than renal tissue. Likewise, it is also noted that TQ does not have any effects on iNOS, TNF-α, and NF-ĸB levels, which are anti-inflammatory genetic factors. Though these parameters are studied on renal tissue, our study has led to different results if compared with the rest of the medical literature. 

In a nephropathy model induced by cisplatin, a 50 mg/L dose of oral TQ was given to rats. The study revealed that TQ makes a positive effect on histopathological parameters (37).The efficacy of TQ was also shown in a nephropathy model where AKI is induced by administration of mercury chloride (HgCl_2_) (17). In that study, when administered in 10 mg/kg/day oral dose, TQ prevented renal histopathological damages. Through creation of a CIN model, TN, PC, and MC generated in renal tissue are examined and assessed in two separate clinic studies (20,44). In these clinical studies where renal protection effects are demonstrated against CM damages through administration of adrenomedulline and recombinant manganese superoxide dismutase, it is emphasized that improvement is achieved in results of histopathological assessments. We have also examined TB, PC, and MC levels again as histopathological damage indicators. According to our results, the contrast nephropathy model created by us (group M) has resulted in deterioration in all of three histopathological parameters (Table 4). It is further understood that TN and PC damages are remedied by low dose TQ treatment (group TA), but MC damage is irreversible by nature. In addition, it is found out that high dose TQ treatment (group TB) does not correct or improve any one of these three histopathological findings, but on the contrary leads to a higher deterioration in comparison to group M. Interestingly, MC damages were observed in kidneys of subjects (group T2) to whom only high dose TQ is administered without creation of the CIN model. In reliance upon these experimental results, we believe that in order to achieve histopathological improvement in subjects with contrast nephropathy, TQ dose to be administered is important, and high dose TQ may further potentialize histopathological deterioration. The finding of MC damage even in the T2 group indicates that high doses of TQ may even alone lead to deterioration in renal histopathology. 

Our study has some restrictive points. First is that the number of rats has been kept low due to a restriction linked to ethical reasons. Again due to technical reasons, we thought that we could not derive reliable data, and therefore, we did not check NGAL level in urine. So as not to lead to any change in volume balance of rats, we have not taken blood samples from rats at the beginning of and during the experiment.

## Conclusion

Prevention of nephrotoxic effects of CM frequently used in daily practice constitutes one of the current subjects of studies. This experimental study has demonstrated for the first time the protective effect of the TQ substance for CIN in 1 mg/kg dose, in the accompaniment of biochemical and histopathological data. Though we could not derive a clear data and conclusion showing that TQ exerts this positive effect through the anti-inflammatory or anti-oxidant pathway, we think that this subject must be dealt with further in the future clinical studies. This means that more clinical studies are needed in order to determine the efficacy and appropriate dose range of TQ in contrast to substance prophylaxis.
